# Disclosure of Pharmaceutical Industry Funding of Patient Organisations in Nordic Countries: Can Industry Self-Regulation Deliver on its Transparency Promise?

**DOI:** 10.1177/00207314221083871

**Published:** 2022-03-01

**Authors:** Dylan Pashley, Piotr Ozieranski, Shai Mulinari

**Affiliations:** 1 Department of Sociology, Faculty of Social Sciences, 5193 Lund University, Lund, Sweden; 2 Department of Social and Policy Sciences, 1555 University of Bath, Bath, UK

## Abstract

Pharmaceutical companies regularly fund patient organizations. It is important for patient organizations’ credibility that there be transparency regarding this financial support. In Europe, the pharmaceutical industry promises to deliver transparency through self-regulation, as opposed to legally binding provisions, but self-regulation's effectiveness is contested. We compared the industry's transparency of funding in four Nordic countries that, given their general reputation for high transparency, offered a critical test of self-regulation's ability to deliver on its transparency promise. For 2017–2019, we compared: national rules regarding funding disclosure; disclosure practices as evidenced by the availability, accessibility, and format of company transparency reports; and disclosure data, including payment descriptions and sums. Transparency problems differed in kind and magnitude between countries. In Norway and Finland, unlike in Sweden and Denmark, data on funding were difficult to access and analyze and sometimes seemed incomplete or missing. We explain that a key factor allowing for country differences is the freedom given to a country's pharmaceutical industry trade associations to form self-regulatory rules, provided they do not fall below the weak, European-level minimum requirements. Transparency could be improved by aligning rules and practices with the FAIR data principles: that is, corporate disclosures should be findable, accessible, interoperable, and reusable.

Patient organizations and pharmaceutical companies cooperate in disease advocacy, lobbying, patient education, and research.^1^ These collaborations often involve companies providing patient organizations with funding,^2^ which is likely to increase their capacity.^3^ At the same time, industry funding may shape patient organizations’ agendas and ideologies^2,4^—for example, prompting some industry-funded organizations to lobby governments for expanded access to novel drugs,^5–9^ rather than to lobby industry to lower drug prices to achieve the same goal.^10,11^ Furthermore, industry sponsorship may weaken patient organizations’ representativeness and credibility^12,13^ and influence which organizations, voices, and diseases get more attention in society.^14^

More than a decade ago, research began to assess the pattern of industry funding of patient organizations,^15–19^ but this effort has been hindered by the paucity of reliable data.^20^ Only more recently have researchers been able to access more complete datasets, disclosed by pharmaceutical companies under self-regulatory frameworks set up by pharmaceutical industry trade associations (PTAs) across countries. So far, single-country studies of Australia,^21^ Sweden,^14^ the United Kingdom,^1,4,6^ and the United States^22^ have used these data to build national pictures of industry–patient organization ties, showing, for example, how funding is concentrated in commercially high-profile disease areas such as cancer, but not in low-profile ones such as mental illness, and how several large companies do most of this concentrated funding.

In Europe, such research is enabled by the codes of the European Federation of Pharmaceutical Industries and Associations (EFPIA): starting in 2011, the Patient Organisation Code of Practice,^23^ and starting in June 2019, the unified EFPIA Code of Practice itself.^24^ The EFPIA Code lays down the *minimum* requirements for companies to follow to “ensure that relationships between the pharmaceutical industry and patient organizations take place in an ethical and transparent manner.” Regarding the transparency of funding, the key provisions are as follows (Article 24):

Each Member Company must disclose a list of POs [Patient Organizations] to which it provides financial support and/or significant indirect/non-financial support or with whom it has engaged to provide contracted services for that Member Company. This disclosure must include a description of the nature of the support or services provided that is sufficiently complete to enable the average reader to form an understanding of the nature of the support or the arrangement without the necessity to divulge confidential information … This information must be disclosed on the Member Company website either on a national or European level on an annual basis and each Reporting Period shall cover a full calendar year.

However, evaluation of funding disclosures in the United Kingdom showed that some companies failed to meet these minimum requirements—for example, by not giving appropriate descriptions of some payments^25^ or even by withholding transparency reports.^26^ However, the Swedish PTA has moved beyond EFPIA's minimum requirements by establishing a centralized, searchable database where companies should upload reports of every payment.^14^ Yet, to date, no systematic research has examined whether or not national PTA rules surpass EFPIA minimum requirements, how they are applied, and to what extent they address the problem of poor transparency that has hampered research in the past.

The timeliness of country comparisons in this area is underlined by insights from international comparisons in related areas of self-regulation, such as drug promotion^27,28^ and the disclosure of payments to healthcare professionals (HCPs).^29,30^ This research has revealed, for example, similar self-regulatory arrangements but different oversight mechanisms between countries.^27^ Furthermore, because self-regulation draws on common drug industry standards (eg, the European-level minimum requirements demanded by EFPIA) to regulate essentially the same set of companies and activities, insights are relatively easily transferable across countries.

Against this background, this study investigates industry disclosures of the funding of patient organizations in the four largest Nordic countries. Significantly, the Nordics are generally known for transparency and openness in policymaking,^31^ including related to pharmaceuticals.^32,33^ They should therefore offer a critical test of the EFPIA self-regulatory framework's ability to ensure transparency. Furthermore, the Nordics are commonly perceived as quite similar when viewed from a broader international perspective, meaning that policies and practices of industry funding and transparency should be comparable and insights readily transferable. The Nordics have relatively similar welfare and political systems, with some observers even speaking of a “Nordic model” of the welfare state,^34^ including health care.^31^ There is also close Nordic political and other cooperation, including in health care, science, civil society, and business.^35^ In the pharmaceutical industry this is reflected, for example, in Nordic PTA cooperation in a range of policy areas, notably including self-regulation and compliance^36^; in some firms having Nordic branches; and in joint Nordic drug industry news outlets and events.^37^

Similarities and collaboration extend to patient organizations. In all four countries, patient organizations have increased in number and influence in recent decades, assuming the role of pressure groups against health care authorities and politicians.^14,38–40^ Moreover, as with PTAs, some collaborate at the Nordic level.^41,42^ In terms of industry funding in the different countries, Hemminki and colleagues^16^ reported that in Finland in 2004, 39 of 55 (71%) surveyed patient organizations acknowledged industry support and that all 20 drug firms said they cooperated with patient organizations. However, the study could not quantify the funding due to the lack of an industry disclosure policy at the time. By contrast, a recent Swedish study^14^ showed that in 2014–2018, 46 companies reported 1412 payments to 77 patient organizations worth at least €6.4 million. To our knowledge, there are no studies of industry sponsorship of patient organizations in Denmark or Norway, but because of the close relations between the Nordics, the basic prediction would be of similarity in policies and practices regarding funding and transparency, although total sums are expected to be higher in Sweden because of its almost twice-as-large population: 10.2 million, versus 5.8 million in Denmark, 5.5 million in Finland, and 5.3 million in Norway.

Below, we show that this basic prediction of similarity is largely *unfounded* by looking at the implementation of industry disclosure requirements in the Nordic countries. More generally, we intend to illustrate, through the prism of the four Nordic countries, the disparate state of transparency in the European pharmaceutical industry. A key factor that allows for country differences is the freedom afforded to each country's PTA in forming and implementing its self-regulatory policy, constrained by the minimum standards laid down by EFPIA—which do not themselves ensure transparency.

## Methods

This study uses three data sources: first, transparency *rules* codified in PTA codes at the European and national levels, as well as any related national transparency legislation; second, disclosure *practices* as evidenced by the accessibility, availability, and format of company transparency reports at the national level; and, third, disclosure *data* extracted from company transparency reports, including data on payment descriptions and sums.

### Disclosure Rules

We reviewed the EFPIA Code and the PTA codes of Denmark, Norway, Finland, and Sweden, valid since 2017, to understand national rules relative to EFPIA minimum requirements. Consistent with previous research on drug industry self-regulatory transparency frameworks,^25,29,30,43,44^ we compared the rules regarding the required or recommended: (*a*) disclosure platform (eg, *centralized* on the trade group website or *decentralized* on company websites); (*b*) information content (eg, recipient names, sums, and descriptions of the funding purpose); (*c*) reporting periods (eg, yearly) and retention of disclosures (eg, three years in the public domain); and (*d*) additional information beyond descriptions of payments (eg, contracts made with patient organizations or methods used by companies when collating reports). In addition, we compared, based on the Codes and associated information on PTA websites: (*e*) the list of companies to which the rules applied in the different countries based on PTA membership; (*f*) the rules regarding company oversight (eg, self-regulatory bodies handling complaints regarding nondisclosure); and (*g*) any national legislation mandating funding disclosure.

### Disclosure Practices

We sought to compare practices in 2017–2019, consistent with the industry's three-year standard of public data retention.^23^ We considered the availability, accessibility, and format of transparency reports, as these are crucial aspects of transparency.^30,44^ For example, having disclosure reports dispersed on individual company websites in formats customized by specific companies severely undermines the possibility of sector-wide analysis of payments. Specifically, for each company in each country, we registered information on whether and how transparency reports were found (eg, via centralized database, out-links on PTA website, or website searches), whether reports were available on corporate national or international websites, and their format (eg, PDFs).

For Sweden and Denmark, where the PTAs provide centralized access to reports, identifying disclosing companies and their reports was straightforward. In contrast, reporting in Norway and Finland is dispersed on company websites, but the PTAs have designated gateways on their websites where companies can add out-links to transparency webpages. The out-links provided the starting point for our manual searches, carried out between February 15 and 27, 2021. If disclosure reports were not found on the linked webpages, website subheadings “transparency,” “support,” and “collaborations” were inspected. For any Norwegian or Finnish PTA member company without out-links on the relevant trade group's gateway, we similarly searched its national, European, and global websites (in that order) for reports. If this failed to identity reports, we resorted to Google searches using the company name connected to keywords similar to the above subheadings in Norwegian and Finnish, respectively.

### Disclosure Data

#### Extraction of Data From Reports

We developed three extraction methods to match the variable disclosure practices across countries. First, for the Swedish reports, which are available in a centralized online database, we designed a web scraper in R^45^ that automatically downloaded the data into a spreadsheet. We scraped data for 2019, which was combined with the 2014–2018 database published by Mulinari and colleagues,^14^ created using manual rather than automatic extraction.

Second, for the Danish reports, which are assembled in one industry-wide PDF spreadsheet per year, we used a mix of manual and automated tabular extraction in the Java program Tabula and in R. Manual work was needed to edit the last row on many pages, because it often extended into the next page, making automated reading difficult.

Third, for Finland and Norway, where reporting formats are not standardized across companies, we manually copied the data from each report, cell by cell, into a spreadsheet with the following headings, consistent with headings used by the industry in Sweden and Denmark: name of company donor; name of recipient; description of the payment; the date or, if unavailable, year of payment; and financial or non-financial information about the value of the support.

For all countries, some manual standardization of the extracted data was needed—for example, correcting the variable uses of period and comma separators when reporting sums. Furthermore, to facilitate comparison, non-euro currencies were converted to euros using daily exchange rates (or annual averages when no date information was reported) available from the “priceR” R package, inflation adjusted to the 2018 euro value.

#### Data Completeness and Quality

Drawing on previous UK research,^25^ we evaluated three key provisions of EFPIA's minimum transparency rules^23^ in the four countries:

1. Availability of reports: “Each Member Company must disclose a list of POs [Patient Organizations] to which it provides financial support and/or significant indirect/non-financial support or with whom it has engaged to provide contracted services for that Member Company.” We assessed the possibility of nondisclosure by comparing the pattern of disclosure at the country and company levels—that is, the number of companies with available reports for all years in each country as well as the total reported value of payments.
2. Informative descriptions: “This disclosure must include a description of the nature of the support or services provided that is sufficiently complete to enable the average reader to form an understanding of the nature of the support or the arrangement without the necessity to divulge confidential information.” We assumed that more words typically equated to more clarity and detail, and we therefore compared the lengths of payment descriptions in the four countries.
3. Monetary information: “In addition to the name of the PO, the following elements must be included: For support: the monetary value of financial support and of invoiced costs.” We calculated the frequency of payment reports in each country and per company that implied financial support but that lacked information on payment sums.

## Results

We first analyze the industry disclosure rules in the four Nordic countries. We then examine their actual application by analyzing the availability, accessibility, format, and content of the transparency reports published by companies in each country.

### Industry Disclosure Rules in the Nordic Countries

Overall, industry rules in Denmark and Sweden, but not in Norway and Finland, clearly go beyond the minimum EFPIA requirements. Table 1 summarizes key similarities and differences across the four countries. Most importantly, there is *centralized* access to summary transparency reports in Denmark (ie, a yearly industry-wide PDF) and Sweden (ie, a regularly updated online database). Companies in Sweden can also add out-links and attachments in the database to more detailed or formal agreements, even though there is no requirement or recommendation from EFPIA to make such agreements public. In Denmark, the rules are even more demanding in this respect, as companies are always obliged to publish, separately, their agreements with patient organizations on their websites “when the agreement is concluded.” Agreements may be more detailed than the description in the industry-wide PDF, but agreements need only be kept online for six months. However, companies in both countries are obliged to make older agreements available to third parties upon request, with no time limit in Sweden and for 10 years in Denmark.

**Table 1.** Similarities and Differences in Industry Disclosure Rules Across the Four Nordic Countries Compared with the EFPIA Code of Practice.

Dimension	Europe	Denmark	Sweden	Finland	Norway
***Organization***	The European Federation of Pharmaceutical Industries and Associations (EFPIA)	Ethical Committee for the Pharmaceutical Industry (ENLI) to whose rules members of the Danish Association of the Pharmaceutical Industry (LIF-DK) are subjected	Swedish Association of the Pharmaceutical Industry (LIF-SWE)	Pharma Industry Finland (PIF)	Association of the Pharmaceutical Industry in Norway (LMI)
***Rule applicable to***	Set minimum standards for national pharmaceutical trade associations (PTAs)	LIF-DK members and trade groups for generic manufacturers and parallel importers of pharmaceuticals	LIF-SWE full and associate members plus members of trade groups for contract research organizations, generic manufacturers, and smaller biotech companies	PIF members	LMI members
***Platform of disclosure***	***Decentralized*** Companies must make available a list of patient organizations to which it provides support or services	***Centralized*** Companies must once a year submit an overview to ENLI covering their collaborative projects. ENLI publishes the summaries on its website ***Decentralized*** Agreements with patient organizations must also be published on company websites	***Centralized*** Companies must make short versions of all contracts and agreements available in the LIF-SWE co-operation online database	***Decentralized*** Companies must maintain an updated and publicly accessible list of the sponsored patient organizations. No mention of website with out-links in industry Code, but PIF hosts such a website	***Decentralized*** Companies must disclose a list containing all patient organizations to which they provide financial and significant non-financial contributions. Companies are obliged to help LMI create a link from a common reporting website
***When disclosures shall be made***	At least updated on annual basis	***Centralized*** Annual basis ***Decentralised*** When the agreement is concluded	When activity is ongoing: in 2020 specified as “at the latest at the time of implementation of the collaboration”	Annual basis	Annual basis
***Retention of records in public domain***	Since 2019: for a minimum of three years	***Centralised*** ENLI website for three years ***Decentralised*** At least six months after termination of the collaboration project	For at least one month after project is concluded, but in practice three years and then automatically unpublished; rules updated in 2020 to reflect this practice	Three years, and must be kept by company for at least five years	Three years, and must be kept by company for at least seven years after the end of the reporting period
***What information shall be disclosed***	Name of patient organization; the monetary value of financial support and of invoiced costs, or the non-monetary benefit; for contracted services: the total amount paid per patient organization over the reporting period, and “must include a description of the nature of the support or services provided that is sufficiently complete to enable the average reader to form an understanding of the nature of the support or the arrangement without the necessity to divulge confidential information”	Name of the collaboration project; name of the parties that have entered into the agreement; types of projects; purpose of the agreement, roles of the parties in the project; timeframe of the project; size of the financial support given and what it is used for; scope and content of non-financial support	Name of the collaboration project; start date and end date; the company; the collaborating party; project description; financial details; additional information (if relevant): contact information; weblinks (if relevant); attachments (if relevant)	A brief description of the nature of the sponsorship and the financial value of the direct or indirect value	The total cost: for significant, non-financial contributions, it must appear clearly what benefits the patient organization received; for assignments, the following must be disclosed: the total fee paid to each patient organization in the period of reporting Suggested disclosure template available from LMI
***Additional rules***	Since 2019, each member company must publish the methodologies used by it in preparing the disclosures and identifying supports and services provided	Copies of the agreements must be made available upon specific request, but not for collaborations that expired more than ten years ago	Contracts and agreements between organizations and pharmaceutical companies shall also be kept available for third parties; openness relates to all agreements, whether ongoing, concluded, or regarding future projects	Companies are “invited to include a clause in their co-operation agreements with patient organisations” stating that the patient organization is obliged to disclose their current or past contractual relationship with the company every time a representative of the organization publicly writes or speaks about an issue focused on by the agreement or otherwise related to the company	Companies must disclose the methods used when publishing and identifying transfers to patient organizations
***National transparency legislation relevant to donors or recipients***	None	***Recipients*** Bekendtgørelse om reklame mv. for lægemidler §21 and Lægemiddelloven § 71 d specify that patient organizations must keep a publicly available record on their websites for a minimum of two years of financial benefits received from pharmaceutical companies, made available at the latest one month after receiving said benefit.	None	***Donors*** Lääkelaki 91 c § specifies that a company that holds a license for the sale of pharmaceutical products shall keep a publicly available updated list of direct and indirect financial support and other comparable support that they have provided to associations in medicine or health care and to patient organizations	None
***Oversight***	None. Each national PTA must establish adequate procedures for ensuring that each of its member companies complies with the PTA's code, including establishing appropriate complaint procedures and sanctions for breaches of their respective codes.	**Self-regulation** Self- regulatory bodies with the task of auditing and assessing any company's violation of the ethical rules can take up complaints, initiate cases, and sanction breaching companies	**Self-regulation** Self-regulatory bodies with the task of auditing and assessing any company's violation of the ethical rules can take up complaints, initiate cases, and sanction breaching companies. LIF-SWE's compliance officer is responsible for quality control of the co-operation database with the aim of working preventatively and supportively, so that the database is kept updated and current	**Self-regulation** Self-regulatory bodies have responsibility for, among other things, co-operation between the industry and patient organizations. The system is based mainly on complaints, and bodies can sanction breaching companies	**Self/co-regulation** LMI in cooperation with the Norwegian Medical Association has set up a self-regulatory body administrated by LMI. The body can take up complaints, initiate cases, and sanction breaching companies

In contrast, companies in Finland and Norway must only keep yearly transparency reports with brief descriptions on their own websites, consistent with EFPIA's minimum requirement. Reports must be stored online for three years, although companies are requested to keep reports for several more years for reference purposes. In Norway, companies are obliged to facilitate the creation of a common gateway by the PTA—that is, a website with out-links to each disclosing company's yearly transparency report, and the same requirement applies to the disclosure of payments to HCPs and healthcare organizations (HCOs), such as hospitals. No similar requirement exists in Finland, but the Finnish PTA has nonetheless created a common gateway on its website.

In Norway, the PTA code also mentions the existence of a disclosure template that companies are encouraged to use to promote more standardized formatting of reports, but the template is not publicly available. Notably, EFPIA has a disclosure template for HCPs and HCOs^24^ but *not* for patient organizations, meaning that companies’ patient organization transparency reports can be expected to be less standardized across countries and companies.

Another important distinction between Denmark/Sweden and Norway/Finland pertains to the list of companies to which the rules apply. PTA members must always comply with the rules,^24^ but in Denmark/Sweden, the rules also apply to companies that are members of certain other, smaller trade associations—for example, generic drug manufacturers. In contrast, only in Finland does transparency legislation require *all* companies to disclose payments irrespective of trade group membership. However, this law, introduced in 2010, does not go beyond the Finnish PTA code in terms of specific disclosure requirements (see Table 1). It only demands that all companies marketing drugs in Finland “shall keep a publicly available updated list of direct and indirect financial support and other comparable support that they have provided to associations in medicine or health care and to patient organisations.” In Denmark, there is also transparency legislation, but it pertains to patient organizations, not companies.

Finally, consistent with EFPIA requirements, all countries have designated industry bodies tasked with handling possible PTA code breaches, including those related to payment disclosure. Specifically, these industry bodies should help identify breaches, investigate complaints against companies, and sanction breaching companies consistent with the codified self-regulatory procedures in each country. However, Sweden's code is unique in that it tasks one individual, the Swedish PTA's Compliance Officer, with responsibility for quality control of transparency reporting in the database.

### Availability, Accessibility, and Format of Transparency Reports in Nordic Countries

We next considered the reporting practices. Forty-seven companies reported payments in Denmark (n = 530 payments; €3,660,705) and 45 in Sweden (n = 874; €3,766,547) in 2017–2019. In contrast, we identified 22 reporting companies in Finland (n = 425; €1,743,001) and 22 in Norway (n = 447; €2,204,051) plus two companies in Norway that stated on their websites that they had no payments to disclose. Table 2 lists all the reporting companies per country.

**Table 2.** Availability of Disclosure Reports from Companies in the Nordic Countries (2017-2019).

Company^a^	Denmark	Sweden	Finland	Norway
AbbVie	2017–2019	2017–2019	2017, 2018	2017–2019
Actelion	2018	2017–2019	Not member^b^	Not member
Akcea	2018, 2019	Not found/no payments^c^	Not member	Not member
ALK Nordic	2017	Not found/no payments	Not member	Not member
Allergan	Not member	2017	Not member	Not member
Almirall	2017, 2018	2018	Not member	Not member
Alnylam	2019	Not found/no payments	Not member	Not member
Amgen	2017	2017–2019	2017–2019	2017–2019
Amicus	2018, 2019	Not found/no payments	Not member	Not member
Astellas	2017–2019	2017–2019	2019	2017–2019
AstraZeneca	2017–2019	2017–2019	2017–2019	2017–2019
Baxalta (acquired by Shire, later by Takeda)	2017	Not member	Not member	Not member
Baxter	Not member	2017	Not member	Not member
Bayer	2017–2019	2017–2019	2017–2019	Not found/no payments
Biogen	2017, 2019	2017–2019	2017–2019	2019
BioMarin	Not member	2019	Not member	Not member
Boehringer Ingelheim	2017–2019	2017–2019	2017–2019	2018, 2019
Bristol Myers Squibb	2017–2019	2017–2019	Not found/no payments	2017, 2019
Campus	Not member	2019	Not member	Not member
Celgene	2017–2019	2017–2019	Not found/no payments	Not member
Chiesi	2018, 2019	2019	Not member	Not found/no payments
CSL Behring	2018	2017–2019	Not member	Not member
Eisai	Not found/no payments	Not found/no payments	Not member	2018
Eli Lilly	2017–2019	2017–2019	2017–2019	2017–2019
Ferring	2017	2019	2019	Not member
GE Healthcare	Not member	Not member	Not member	Nothing to disclose
Gilead	2018, 2019	2017–2019	Not member	Not member
GlaxoSmithKline	2017–2019	2017–2019	2019	2017–2019
Grünenthal	2018, 2019	2019	Not member	Not found/no payments
Indivior	2019	Not member	Not member	Not member
IPSEN	2017–2019	2017–2019	Not member	Not member
Janssen-Cilag	2017–2019	2017–2019	2017–2019	2017–2019
Kyowa Kirin	2019	2019	Not member	Not member
LEO Pharma	2017–2019	2019	2018, 2019	Not found/no payments
Lundbeck	2017–2019	Not found/no payments	2017–2019	Not found/no payments
Merck	2017–2019	2017–2019	2017–2019	2017–2019
MSD	2017–2019	2017–2019	2018, 2019	2017–2019
Mundipharma	2017	Not member	Not member	Not found/no payments
Nigaard	Not member	2017	Not member	Not member
Nordic Drugs	Not member	2018, 2019	Not member	Not member
Nordic Infu Care	2019	Not member	Not member	Not member
Norgine	2018, 2019	2019	Not member	Not member
Novartis	2017–2019	2017–2019	2017–2019	2017–2019
Novo Nordisk	2017–2019	2017–2019	Not found/no payments	2019
Octapharma	2018, 2019	2017, 2019	Not member	Not member
Orifarm	2017	Not found/no payments	Not member	Not member
Orion	Not found/no payments	Not found/no payments	Not member	Nothing to disclose
Otsuka	2017, 2019	2017–2019	Not member	2017
Pfizer	2017–2019	2017–2019	2017–2019	2017–2019
Pierre Fabre	2017	Not found/no payments	Not member	Not found/no payments
Roche	2017–2019	2017–2019	2019	2019
Sanofi	2017–2019	2017–2019	Not found/no payments	2019
Servier	Not member	2019	Not found/no payments	Not found/no payments
Shire	2018, 2019	2017–2019	2019	2019
Swedish Orphan Biovitrum (SOBI)	Not member	2017, 2019	2017–2019	Not member
Takeda	2017–2019	2017–2019	2019	2017–2019
Teva	2017	2018, 2019	Not member	Not member
Tillotts	Not member	2017, 2019	Not member	Not member
UCB	2017–2019	2017, 2019	2017–2019	2017–2019
**N companies, at least one year**	**47**	**45**	**22**	**24**
**N companies, all years**	**22**	**26**	**13**	**15**

^a^ Company included in list if reports were found for one or more countries.

^b^ Not member = Not listed as Pharmaceutical Trade Association member.

^c^ Not found/no payments = Listed as Pharmaceutical Trade Association member but no reports found.

The difference between Denmark/Sweden and Finland/Norway may at least partly be due to more companies in Denmark/Sweden committing themselves to following the PTA codes. The Swedish PTA lists 99 companies on its website that are either full members (pharmaceutical companies) or associate members (clinical research organizations and smaller biotech companies). The Norwegian PTA lists 61 members. In contrast, the Danish and Finnish PTAs list only 36 and 39 members, respectively, most of which are big global pharmaceutical companies. Nonetheless, the four PTAs’ membership lists largely overlap regarding the 20 largest companies worldwide^46^: all are members in Sweden, but Gilead and Teva are not members in the other countries, although Teva is a member of the Danish generic manufacturers’ association.

The difference may also be due to less industry–patient group interaction in Finland/Norway. We compared the pattern of payments from the six companies from which we found payment reports for all years in all the countries: AstraZeneca, Eli Lilly, Janssen, Merck, Novartis, and Pfizer. Together, these large companies reported similar amounts in all countries—€1,020,710 in Denmark, €1,187,704 in Sweden, €904,531 in Finland, and €1,291,960 in Norway—suggesting a similar appetite for making payments in each country.

At the company level, unavailable transparency reports from PTA members could indicate that no payments were made, but it can also indicate that companies, in violation of the rules, either failed to disclose altogether, disclosed in an inaccessible location or format, or removed their reports prematurely.^1^ Supplementary Table 1 details the availability, accessibility, and format of transparency reports of PTA members in Norway. The Norwegian PTA gateway contained links to disclosures from 36 of 61 members. Only three companies (AstraZeneca, Eli Lilly, and Pfizer) had their out-links labeled to signal that disclosures pertained to patient organizations. Twenty-two of 31 companies (61.1%) had their links labeled “Disclosure of Transfers of Value.” Seven (31.8%) linked to a webpage with at least one disclosure pertaining to a Norwegian patient organization. The remaining 11 of 36 companies (30.6%) had their links labeled “Transfers of Value HCP/HCO [Healthcare Professional/Healthcare Organization],” signaling that these companies had no patient organization disclosures. Yet, for four companies (AbbVie, Biogen, Eisai, and GE Healthcare), these out-links led to company webpages that *did* contain disclosures pertaining to patient organizations. Altogether, we were able to access at least one Norwegian patient organization transparency report from each of 15 companies via the PTA gateway. However, website and Google searches identified reports from nine more companies, all of which were listed on the PTA gateway, resulting in a total of 24 disclosing companies, two of which (GE Healthcare and Orion) stated that they had no payments to disclose. Seventeen (70.1%) companies published their reports as customized PDFs, with the rest having either online text or databases on their websites, either as part of Norwegian or international disclosures. Fifteen (62.5%) companies had transparency reports for all years, including GE Healthcare and Orion, with the rest, including several large companies, disclosing in one (n = 7) or two (n = 2) years.

Supplementary Table 2 shows the availability, accessibility, and format of transparency reports in Finland. The Finnish PTA gateway contained out-links labeled with company names. These out-links led to disclosures from 30 of 39 members; only nine (30.0%) led to patient organization disclosures, whereas the remaining 21 led to HCP/HCO disclosures only. However, website and Google searches identified patient organization transparency reports from 13 more companies, all of which were listed on the PTA gateway, resulting in a total of 22 disclosing companies. Ten (45.5%) published transparency reports as customized PDFs and, notably, two large companies (AstraZeneca and Boehringer Ingelheim) made their patient organization disclosures in conjunction with HCP/HCO disclosures. This contrasts with the inference from the self-regulatory requirement that patient organization and HCP/HCO disclosures should be kept separate to ensure compliance with the PTA rules governing their respective disclosures. The rest had either online text or databases on their websites, either as part of Finnish or international disclosures. Thirteen of the 22 companies (59.1%) had disclosure reports for all years in Finland, with the rest disclosing in one (n = 6) or two (n = 3) years. As in Norway, this latter group included several large companies.

### Length of Descriptions in Transparency Reports

**Figure 1.**
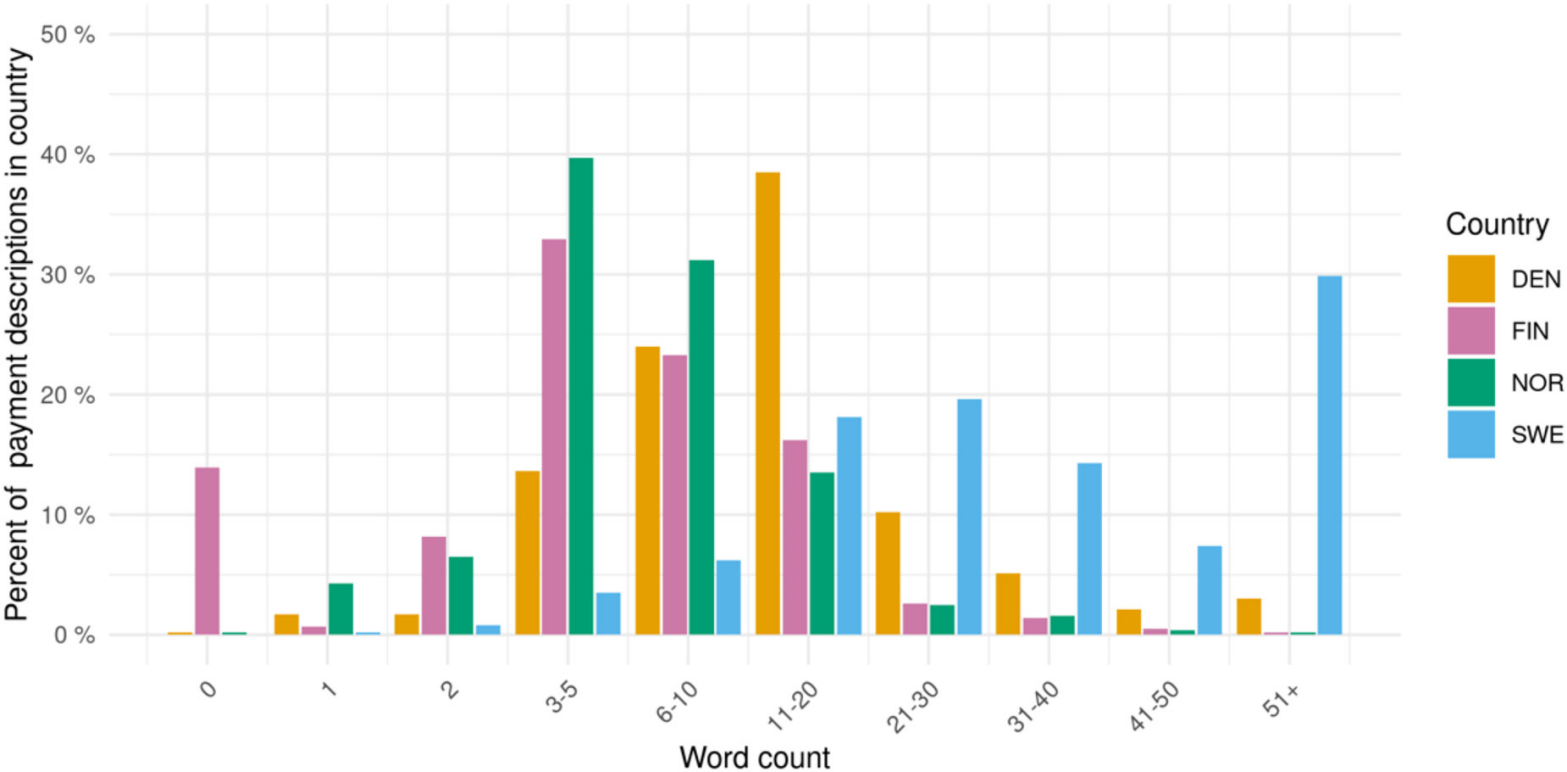
Words used in companies’ patient organization payment descriptions in the nordic countries, 2017–2019.

The histogram in Figure 1 shows the number of words used in individual payment descriptions in the Nordic countries. Finnish and Norwegian descriptions were shorter: Just under 50% have more than five words and fewer than 5% have more than 20 words, in both countries. This contrasted with Denmark, where 82.8% had more than five words and 20.4% more than 20 words, and especially with Sweden, where 95.4% had more than five words, 71.2% had more than 20 words, and a sizable proportion, around 30%, even had more than 50 words. Table 3 shows selected examples of descriptions of different lengths from one company (Roche) in the four countries in 2019.

**Table 3.** Selected Examples of Roche's Payment Descriptions of Different Lengths in 2019.

Word length^a^	Country	Sum (€)	Description
3	FIN	1582.8	Grant advocacy workshops
5	NOR	15,146.2	Financial contributions to patient organizations
23	DEN	10,621.7	Economic support for qualitative evaluation of the use of the Floodlight app in the Tension and Trauma Releasing Exercises (TRE) research project.
51	SWE	Not specified: “Roche covers the printing costs; the [funded] association covers the labour costs.”	Roche Ltd in collaboration with Boehringer Ingelheim and the Swedish Association for Pulmonary Fibrosis wishes to draw attention to the disease area by producing an IPF [ie, idiopathic pulmonary fibrosis] report that can be used by the Association and the pharmaceutical companies. The hope is also that *DN* [ie, a major Swedish newspaper] will be able to write a story that highlights the disease and its problems, to stimulate debate and attract attention.
72	SWE	Not specified: “Roche Ltd has agreed to bear 50% of the costs incurred. The Foundation has agreed to bear the remaining 50% of the costs incurred.”	The last Wednesday in May every year is International MS [ie, multiple sclerosis] Day. It was launched on 27 May 2009 with over 200 events in 67 countries. The aim is to increase awareness of MS and strengthen the network of people living with MS around the world. In 2019, Roche and the Foundation will jointly arrange the meeting, which will take place in Stockholm on 27 May 2019. Roche and the Foundation will set the agenda, book a venue, invite moderators and relevant speakers, as well as market the event.
119	SWE	5916.3	The Swedish Blood Cancer Association has initiated the project “The road to a deep treatment response” (VTEDB). A [special] interest-group–driven initiative that spans the entire country will consist of three closed roundtable talks that will take place in Stockholm, Lund, and Umeå. Furthermore, VTEDB will focus on a single issue, which is access to the best treatment, which will be concretized with the example of how to achieve a deep treatment response. The main target group of the project is political representatives, who have the mandate for effecting change. The aim is that patients will ultimately be able to access modern treatments in a faster and more equal way across the country. Dates for roundtable discussions are: Skåne [ie, southernmost province] on 20 March, Norrland [ie, northernmost geographic region] on 1 April, and central Sweden on 9 May, as well as a seminar during Almedalen Week [ie, annual politicians’ week, see^6^].
			

^a^ Length was calculated from descriptions prior to any translation into English.

In Finland, 12.8% of individual reports (from a total of six companies) did not contain any descriptions. Many of these missing descriptions were associated with companies’ use of inappropriate disclosure formats, including from two companies that disclosed payments to patient organizations in combination with HCP/HCO disclosures for which no individual payment descriptions are required.

### Completeness of Monetary Information in Transparency Reports

Forty-five payments (5.1%) from 15 companies in Sweden lacked payment amounts, excluding any payments reported as zero (ie, non-financial support) (see Supplementary File 1); 20 (44.4%) came from one company, Roche. Reading the descriptions of these payments suggested that many pertained to costs of events (see, eg, Table 3), travel, and hospitality. We did not identify missing monetary information in the other countries.

## Discussion

In this first cross-national study of industry transparency regarding the funding of patient organizations, we considered four Nordic countries that, given their reputation for transparency, offered a critical test of self-regulation's capacity to deliver on its transparency promise. We found transparency problems in all four countries, but they were of a different sort and magnitude in Norway and Finland, where data on funding were difficult to access and analyze, and some data seemed to be missing or failed to comply with the minimum self-regulatory rules regarding information content.

Across the four countries, between 2017 and 2019, we identified 2276 payments, totaling around €11.4 million. Roughly one third of these payments were reported in each of Denmark and Sweden, while the combined funding in Finland and Norway accounted for the remaining one third. However, our analysis raised the suspicion that some companies in Finland and Norway withheld reports. Disclosures from the six companies that did report funding in all four countries during the study period suggested a similar appetite for funding patient groups in the four countries. If this appetite can be extrapolated to the industry as a whole, this would suggest that about 40–50% of the totals were unaccounted for in Finland and Norway. However, it is impossible to determine with certainty the existence, let alone the extent, of missing reports. This is because there is no definitive list of all possible companies that are funding patient organizations and no policy compelling companies to report that they have no funding to disclose, if that is indeed the case, which would resolve any ambiguity regarding the reason for absent reports.

Importantly, the suspicion of absent reports from some companies in Norway and Finland was associated with the lack of a centralized disclosure platform, consistent with the similar experience in the United Kingdom.^4,25,26^ For example, 24 pharmaceutical companies, almost 40% of the total number, mentioned by UK patient organizations as donors in 2012–2016 did not disclose any payments.^26^ From the perspective of transparency, it is therefore helpful that Norwegian and Finnish PTAs have set up gateways that should allow access to companies’ disclosure websites. However, the link to the gateway within the Norwegian PTA's website is labeled “Transfers of Value to Healthcare Professionals [Verdioverføringer til helsepersonell].” Furthermore, several companies labeled their out-links “Transfers of Value HCP/HCO,” even though the out-links led to websites with transparency reports for patient organizations as well. Finally, in both countries, for some companies whose out-links led to HCP/HCO disclosures but not patient organization disclosures, separate website or Google searches did find patient organization disclosures.

The transparency problems in Finland and Norway were not limited to suspicions of missing reports, or to ambiguities regarding whether and how to search for reports. There were also problems related to the disparate formats and low quality of the transparency reports. First, the disparate formats mean that considerable effort is needed to understand the reports, and it also makes data extraction laborious even for small countries such as Finland and Norway. Second, regarding quality, many reports contain only a few words describing the purpose of the funding, and in Finland an important share (13%) lacked descriptions altogether. A similar analysis of the United Kingdom found that only 0.6% of reports lacked descriptions, underscoring the magnitude of the problem in Finland.^25^ Uninformative descriptions also seem recurrent in Denmark, but it is possible that more detailed reports exist on company websites. However, because these reports are only mandated to be kept online for six months, this does not ensure that companies will comply with the transparency rules, which require that reports with informative descriptions should be kept in the public domain for at least three years.

Sweden was the country with the longest descriptions, although about 5% of Swedish records lacked monetary information. We noted that many of the records that lacked information came from one company, Roche, suggesting a systematic problem. It is possible that the lack of information is because some companies upload records soon after an agreement has been reached, at which time the exact value of the funding is unknown. However, failure to update records with monetary information constitutes a rule violation and should be penalized by self-regulatory bodies. To help Swedish self-regulatory bodies, we have compiled a list of relevant cases of missing monetary information (Supplementary File 1).

In light of the problems identified, we conclude that the industry's self-regulation mechanisms are currently insufficient. A key argument for self-regulation is that companies have a strong incentive to monitor competitors’ compliance and complain about code breaches, ensuring that breaching behavior will be rare and transient.^47^ Our results do not support this argument; instead, our study adds to the growing evidence that self-regulation has failed to ensure transparency and compliance throughout the pharmaceutical industry in Europe^26–30,43,44,48–50^ and internationally.^21,51^ More specifically, our study suggests that the major discretion awarded by EFPIA to countries’ PTAs and then by PTAs to companies, together with inadequate oversight, has allowed disparate transparency practices across the industry. In turn, these disparities make investigating the industry funding of patient organizations a daunting task even for a skilled researcher, let alone a member of the public. In particular, it means that payments are practically impossible to analyze in Finland and Norway from a *recipient* perspective (“Who funds this patient organization and why?”). Problems of disparate disclosure practices, including cases of missing or incomplete data, were also highlighted for disclosure of payments to HCPs across a number of European countries.^30^ However, the problems can be expected to be *even greater* for the disclosure of patient organization funding because, unlike in the case of disclosure of payments to HCPs (and HCOs), EFPIA has yet to create a disclosure template or even *try* to establish a minimum level of standardization across Europe.

We speculate that the observed differences between the Nordic countries have their proximal cause in the greater commitment of PTAs in Sweden and Denmark to transparent interaction with patient groups. Consistent with this, we observe that Swedish and Danish PTA codes are more detailed in this area than their Norwegian and, especially, Finnish counterparts. The latter does not even have a separate code section regarding disclosure of payments to patient organizations, instead combining it with the rules regarding disclosure of payments to HCPs/HCOs. As a first step, we would recommend that the Finnish and Norwegian industries adopt similar levels of detail as their Danish and Swedish counterparts and that they implement a centralized disclosure system. However, even disclosure practices in Sweden and Denmark are sub-optimal because preparing the data for analysis required, for Sweden, development of a script to scrape the website and, for Denmark, a combination of automated and manual work to extract the data. By shifting the responsibility for data extraction onto data users, the industry is taking a data quality risk because data extraction may be prone to error or even failure.^30^

Going forward, to enhance transparency and raise standards consistently across Europe, we suggest that EFPIA should adopt the high-level FAIR—findable, accessible, interoperable, and reusable—guiding principles of data management and stewardship.^52^ This would align the EFPIA Code with a number of recent high-profile E.U.^53^ and pharmaceutical industry^54^ initiatives promoting FAIR data principles in areas such as research and development to improve transparency, accountability, and productivity. Briefly, these principles mandate that metadata and data should be easy to *find* for both humans and computers. It should be clear to the user how to *access* the metadata and data. The user should also be able to integrate the metadata and data with other relevant data (eg, through unique identifiers shared across datasets); for this, the data need to *interoperate* with applications or workflows for analysis, storage, and processing. Finally, *reuse* of data—for example, in research—requires that it should be consistently and richly described. As our analysis shows, the current rules and practices of the European pharmaceutical industry fail in each of the FAIR domains.

### Limitations

Our study has several limitations. First, although we argue that companies break the PTA rules when, for example, they do not report payment sums or provide overtly short descriptions, it is possible that self-regulatory bodies interpret these instances differently. Second, despite our comprehensive search strategy, it is possible that some disclosure reports existed but were not found. However, the PTA codes make clear that reports should be publicly disclosed, so if we failed to find existing reports, more than anything this shows that companies are not transparent about patient organization funding. In fact, we believe that our study overemphasizes the transparency in Norway and Finland, because some reports were not easy to find. Third, as noted above, we cannot be certain that unavailable reports were withheld in Norway and Finland; it might be that companies have made no payments and therefore have nothing to disclose. Relatedly, even for companies with available reports, we cannot be certain that these were complete and accurate. Fourth, it is also possible that some of the differences between Denmark/Sweden and Finland/Norway may partly be because more or different companies were PTA members or agreed to abide by the industry code. However, the membership lists of the four trade groups largely overlap when it comes to the 20 largest companies worldwide, which can be assumed to be responsible for most funding based on the present and prior research.^1,14^ Fifth, some companies may have chosen not to abide by the industry code in any of the countries and therefore do not report payments; if so, this means that we probably overestimated transparency and underestimated total funding. Finally, although prior research confirms that more words typically equates to more clarity regarding the purpose of the funding,^25^ ultimately a content analysis of descriptions is needed to confirm this for the Nordic countries as well.

## Conclusion

It is important for the credibility of patient organizations that there is transparency regarding their financial ties to companies. Furthermore, it is important for researchers, policymakers, and the public to be able to identify vested interests that may be influencing patient organizations’ advocacy. Since 2011, the pharmaceutical industry in Europe has promised to deliver transparency through self-regulation, but after 10 years, the industry is falling short of its promise in the Nordic countries. If self-regulation is not delivering transparency in the “easy” Nordic case, it is unlikely to deliver elsewhere. However, the situation is significantly better in Sweden and Denmark, that is, the countries with centralized disclosure, compared with Norway and Finland, that is, the countries relying on decentralized disclosure on company websites, which is completely unsatisfactory from the perspective of transparency. Overall, the limited transparency in Finland is particularly problematic and surprising, because there is a Finnish law mandating disclosure. Yet, even in Sweden and Denmark there is room for improvement, including bettering the oversight to ensure that no data are missing, that disclosure reports contain enough detail, and that the reports can be effectively downloaded and analyzed. We have proposed that aligning disclosures with FAIR data principles could resolve much of the transparency problems still characterizing the interaction between companies and patient organizations.

## Supplemental Material

sj-docx-1-joh-10.1177_00207314221083871 - Supplemental material for Disclosure of Pharmaceutical Industry Funding of Patient Organisations in Nordic Countries: Can Industry Self-Regulation Deliver on its Transparency Promise? Click here for additional data file. Supplemental material, sj-docx-1-joh-10.1177_00207314221083871 for Disclosure of Pharmaceutical Industry Funding of Patient Organisations in Nordic Countries: Can Industry Self-Regulation Deliver on its Transparency Promise? by Dylan Pashley, Piotr Ozieranski and Shai Mulinari in International Journal of Health Services

sj-docx-2-joh-10.1177_00207314221083871 - Supplemental material for Disclosure of Pharmaceutical Industry Funding of Patient Organisations in Nordic Countries: Can Industry Self-Regulation Deliver on its Transparency Promise? Click here for additional data file. Supplemental material, sj-docx-2-joh-10.1177_00207314221083871 for Disclosure of Pharmaceutical Industry Funding of Patient Organisations in Nordic Countries: Can Industry Self-Regulation Deliver on its Transparency Promise? by Dylan Pashley, Piotr Ozieranski and Shai Mulinari in International Journal of Health Services

sj-ods-3-joh-10.1177_00207314221083871 - Supplemental material for Disclosure of Pharmaceutical Industry Funding of Patient Organisations in Nordic Countries: Can Industry Self-Regulation Deliver on its Transparency Promise? Click here for additional data file. Supplemental material, sj-ods-3-joh-10.1177_00207314221083871 for Disclosure of Pharmaceutical Industry Funding of Patient Organisations in Nordic Countries: Can Industry Self-Regulation Deliver on its Transparency Promise? by Dylan Pashley, Piotr Ozieranski and Shai Mulinari in International Journal of Health Services

sj-docx-4-joh-10.1177_00207314221083871 - Supplemental material for Disclosure of Pharmaceutical Industry Funding of Patient Organisations in Nordic Countries: Can Industry Self-Regulation Deliver on its Transparency Promise? Click here for additional data file. Supplemental material, sj-docx-4-joh-10.1177_00207314221083871 for Disclosure of Pharmaceutical Industry Funding of Patient Organisations in Nordic Countries: Can Industry Self-Regulation Deliver on its Transparency Promise? by Dylan Pashley, Piotr Ozieranski and Shai Mulinari in International Journal of Health Services

sj-docx-5-joh-10.1177_00207314221083871 - Supplemental material for Disclosure of Pharmaceutical Industry Funding of Patient Organisations in Nordic Countries: Can Industry Self-Regulation Deliver on its Transparency Promise? Click here for additional data file. Supplemental material, sj-docx-5-joh-10.1177_00207314221083871 for Disclosure of Pharmaceutical Industry Funding of Patient Organisations in Nordic Countries: Can Industry Self-Regulation Deliver on its Transparency Promise? by Dylan Pashley, Piotr Ozieranski and Shai Mulinari in International Journal of Health Services

## References

[1] Ozieranski P Rickard E Mulinari S . Exposing drug industry funding of UK patient organisations. Br Med J. 2019;365(8200):l1806. 31122928 10.1136/bmj.l1806 PMC6529850

[2] Parker L Fabbri A Grundy Q , et al. “Asset exchange”—interactions between patient groups and pharmaceutical industry: australian qualitative study. Br Med J. 2019;367:l6694. 31831471 10.1136/bmj.l6694 PMC7190020

[3] Roennow A Sauvé M Welling J , et al. Collaboration between patient organisations and a clinical research sponsor in a rare disease condition: learnings from a community advisory board and best practice for future collaborations. BMJ Open. 2020;10(12):e039473. 10.1136/bmjopen-2020-039473 PMC7745690 33328257

[4] Ozieranski P Pitter JG Rickard E , et al. A ‘patient–industry complex’? Investigating the financial dependency of UK patient organisations on drug company funding. Sociol Health Illn. 2022;44(1):188–210. 10.1111/1467-9566.13409 34874566

[5] Batt S . Health Advocacy Inc: How Pharmaceutical Funding Changed the Breast Cancer Movement. UBC Press; 2017.

[6] Mandeville KL Barker R Packham A , et al. Financial interests of patient organisations contributing to technology assessment at england’s national institute for health and care excellence: policy review. Br Med J. 2019(8083);364:k5300. 30651227 10.1136/bmj.k5300 PMC6334181

[7] Lexchin J . Association between commercial funding of Canadian patient groups and their views about funding of medicines: an observational study. PLOS ONE. 2019;14(2):e0212399. 30768629 10.1371/journal.pone.0212399 PMC6377138

[8] Batt S Butler J Shannon O , et al. Pharmaceutical ethics and grassroots activism in the United States: a social history perspective. J Bioeth Inq. 2020;17(1):49–60. 31953647 10.1007/s11673-019-09956-8

[9] Marks JH . Lessons from corporate influence in the opioid epidemic: toward a norm of separation. J Bioeth Inq. 2020;17(2):173–189. 32661741 10.1007/s11673-020-09982-x PMC7357445

[10] Committee on Oversight and Reform U.S. House of Representatives. Drug Pricing Investigation AbbVie—Humira and Imbruvica. Washington; 2021. https://oversight.house.gov/sites/democrats.oversight.house.gov/files/Committee%20on%20Oversight%20and%20Reform%20-%20AbbVie%20Staff%20Report.pdf. Accessed January 10, 2022.

[11] Grant K . *How pharma companies try to use funding to sway patient advocate groups*. The Globe and Mail. 2018; October 21. https://www.theglobeandmail.com/canada/article-how-pharma-companies-try-to-usefunding-to-sway-patient-advocate/ Accessed January 10, 2021.

[12] Mintzes B . Should patient groups accept money from drug companies? No. Br Med J. 2007(7600);334:935. 17478846 10.1136/bmj.39185.394005.AD PMC1865416

[13] Moynihan R Bero L . Toward a healthier patient voice: more independence, less industry funding. JAMA Intern Med. 2017;177(3):350–351. 28114596 10.1001/jamainternmed.2016.9179

[14] Mulinari S Vilhelmsson A Rickard E Ozieranski P . Five years of pharmaceutical industry funding of patient organisations in Sweden: cross-sectional study of companies, patient organisations and drugs. PLoS One. 2020;15:e0235021. 32579571 10.1371/journal.pone.0235021 PMC7313941

[15] Baggott R Allsop J Jones KL . Speaking for Patients and Carers: Health Consumer Groups and the Policy Process. Palgrave Macmillan; 2005.

[16] Hemminki E Toiviainen HK Vuorenkoski L . Co-operation between patient organisations and the drug industry in Finland. Soc Sci Med. 2010;70(8):1171–1175. 20163903 10.1016/j.socscimed.2010.01.005

[17] Colombo C Mosconi P Villani W Garattini S . Patient organizations’ funding from pharmaceutical companies: is disclosure clear, complete and accessible to the public? An Italian survey. PLoS One. 2012;7(5):e34974. 22590498 10.1371/journal.pone.0034974 PMC3348919

[18] O’Donovan O . Corporate colonization of health activism? Irish health advocacy organizations’ modes of engagement with pharmaceutical corporations. Int J Health Serv*.* 2007; 37(4):711–733. 18072317 10.2190/HS.37.4.h

[19] Ball DE Tisocki K Herxheimer A . Advertising and disclosure of funding on patient organisation websites: a cross-sectional survey. BMC Public Health. 2006;6:201. 16887025 10.1186/1471-2458-6-201 PMC1557495

[20] Fabbri A Parker L Colombo C , et al. Industry funding of patient and health consumer organisations: systematic review with meta-analysis. Br Med J. 2020;368(8230):16925. 10.1136/bmj.l6925 PMC7190040 31969320

[21] Fabbri A Swandari S Lau E , et al. Pharmaceutical industry funding of health consumer groups in Australia: a cross-sectional analysis. Int J Health Serv. 2019;49(2):273–293. 30646806 10.1177/0020731418823376

[22] Kang S-Y Bai G Karas L Anderson GF . Pharmaceutical industry support of US patient advocacy organizations: an international context. Am J Pub Health. 2019;109(4):559–561. 30789768 10.2105/AJPH.2018.304946 PMC6417565

[23] EFPIA. EFPIA Code of Practice on relationships between the pharmaceutical industry and Patient Organisations. European Federation of Pharmaceutical Industries and Associations; 2011.

[24] EFPIA. The EFPIA Code of Practice. European Federation of Pharmaceutical Industries and Associations; 2019.

[25] Rickard E Ozieranski P Mulinari S . Evaluating the transparency of pharmaceutical company disclosure of payments to patient organisations in the UK. Health Policy. 2019;123(12):1244–1250. 31455562 10.1016/j.healthpol.2019.08.007

[26] Ozieranski P Csanádi M Rickard E Mulinari S . Under-reported relationship: a comparative study of pharmaceutical industry and patient organisation payment disclosures in the UK (2012–2016). BMJ Open. 2020;10(9):e037351. 10.1136/bmjopen-2020-037351 PMC7511620 32950962

[27] Zetterqvist AV Merlo J Mulinari S . Complaints, complainants, and rulings regarding drug promotion in the United Kingdom and Sweden 2004–2012: a quantitative and qualitative study of pharmaceutical industry self-regulation. PLoS Med. 2015;12:e1001785. 25689460 10.1371/journal.pmed.1001785 PMC4331559

[28] Mulinari S Davis C Ozieranski P . Failure of responsive regulation? Pharmaceutical marketing, corporate impression management and off-label promotion of enzalutamide in Europe. JWCC. 2021;2(2):69–80.

[29] Fabbri A Santos A Mezinska S , et al. Sunshine policies and murky shadows in Europe: disclosure of pharmaceutical industry payments to health professionals in nine European countries. Int J of Health Policy Mang. 2018;7(6):504–509. 10.15171/ijhpm.2018.20 PMC6015505 29935127

[30] Mulinari S Martinon L Jachiet PA Ozieranski P . Pharmaceutical industry self-regulation and non-transparency: country and company level analysis of payments to healthcare professionals in seven European countries. Health Policy. 2021;125(7):915–922. 34006392 10.1016/j.healthpol.2021.04.015

[31] Magnussen J Vrangbaek K Saltman R . Nordic Health Care Systems: Recent reforms and current policy challenges. McGraw-Hill Education; 2009.

[32] Skoog M Saarimäki J Gluud C , et al. Transparency and registration in clinical research in the Nordic countries. Nordic Trial Alliance. NordForsk; 2015.

[33] Abraham J Lewis G . Regulating Medicines in Europe: Competition, Expertise and Public Health. Routledge; 2000.

[34] Esping-Andersen G . The Three Worlds of Welfare Capitalism. Princeton University Press; 1990.

[35] Strang J . Nordic Cooperation: A European Region in Transition. Routledge; 2015.

[36] LIF, LMI, PIF, ENLI, FRUMTOK. Nordic Compliance – overview. 2020.

[37] LIF, LMI, PIF, LIF. Kan mer samarbete stärka Life Science i Norden? 2021.

[38] Winblad U Ringard Å . Meeting rising public expectations: the changing roles of patients and citizens. In: J Magnussen, K Vrangbæ, RB Saltman, eds. *Nordic Health Care Systems: Recent Reforms and Current Policy Challenges*. New York: McGraw-Hill/Open University Press; 126–150.

[39] Opedal S Rommetvedt H Vrangbæk K . Organised interests, authority structures and political influence: Danish and Norwegian patient groups compared. Scand Political Stud. 2012;35(1):1–21.

[40] Toiviainen HK Vuorenkoski LH Hemminki EK . Patient organizations in Finland: increasing numbers and great variation. Health Expect. 2010;13(3):221–233. 20579124 10.1111/j.1369-7625.2008.00499.x PMC5060534

[41] Prostatacancerförbundet S Prostatakræftforeningen PROPA D Prostatakreftforeningen PROFO N , et al. Ny nordisk undersökning, världens största i sitt slag, kartlägger den vanligaste typen av cancer bland män. 2014.

[42] European Patient Forum. Nordic Meeting for Primary Immunodeficiency: the shortage of immunoglobulins is a global problem. 2019.

[43] Mulinari S Ozieranski P . Disclosure of payments by pharmaceutical companies to healthcare professionals in the UK: analysis of the association of the British pharmaceutical industry’s disclosure UK database, 2015 and 2016 cohorts. BMJ Open. 2018;8(10):e023094. 10.1136/bmjopen-2018-023094 PMC6196800 30344175

[44] Ozieranski P Martinon L Jachiet PA Mulinari S . Accessibility and quality of drug company disclosures of payments to healthcare professionals and organisations in 37 countries: a European policy review. BMJ Open. 2021;11(12):e053138. 10.1136/bmjopen-2021-053138 PMC8679071 34916317

[45] R Core Team. R: A language and environment for statistical computing; 2017.

[46] Pharmaceutical Executive. Pharm Exec’s Top 50 Companies; 2018.

[47] Francer J Izquierdo JZ Music T , et al. Ethical pharmaceutical promotion and communications worldwide: codes and regulations. Philos Ethics Humanit Med. 2014;9:7. 24679064 10.1186/1747-5341-9-7 PMC3974225

[48] Vilhelmsson A Davis C Mulinari S . Pharmaceutical industry off-label promotion and self-regulation: a document analysis of off-label promotion rulings by the United Kingdom prescription medicines code of practice authority 2003–2012. PLoS Med. 2016;13:e1001945. 26812151 10.1371/journal.pmed.1001945 PMC4727894

[49] Moriarty F Larkin J Fahey T . Payments reported by the pharmaceutical industry in Ireland from 2015 to 2019: an observational study. Health Policy. 2021;125(10):1297–1304. 34429238 10.1016/j.healthpol.2021.07.016

[50] Stoll M Hubenschmid L Koch C Lieb K . Voluntary disclosures of payments from pharmaceutical companies to healthcare professionals in Germany: a descriptive study of disclosures in 2015 and 2016. BMJ Open. 2020;10(9):e037395. 10.1136/bmjopen-2020-037395 PMC7500304 32948560

[51] Ozaki A Saito H Senoo Y , et al. Overview and transparency of non-research payments to healthcare organizations and healthcare professionals from pharmaceutical companies in Japan: analysis of payment data in 2016. Health Policy. 2020;124(7):727–735. 32439213 10.1016/j.healthpol.2020.03.011

[52] Wilkinson MD Dumontier M Aalbersberg IJ , et al. The FAIR guiding principles for scientific data management and stewardship. Sci Data. 2016;3:160018. 26978244 10.1038/sdata.2016.18 PMC4792175

[53] Collins S Genova F Harrower N , et al. Turning FAIR into reality: Final report and action plan from the European Commission expert group on FAIR data. Publications Office of the European Union; 2018.

[54] Wise J de Barron AG Splendiani A , et al. Implementation and relevance of FAIR data principles in biopharmaceutical R&D. Drug Discov Today. 2019;24(4):933–938. 30690198 10.1016/j.drudis.2019.01.008

