# Supplementary material for: Disclosure of Pharmaceutical Industry Funding of Patient Organisations in Nordic Countries: Can Industry Self-Regulation Deliver on its Transparency Promise?
Source: Int J Health Serv. 2022 Mar 1;52(3):347–62. doi: 10.1177/00207314221083871 (PMC9203660; doi:10.1177/00207314221083871)
Supplement: sj-docx-4-joh-10.1177_00207314221083871 - Supplemental material for Disclosure of Pharmaceutical Industry Funding of Patient Organisations in Nordic Countries: Can Industry Self-Regulation Deliver on its Transparency Promise? [file sj-docx-4-joh-10.1177_00207314221083871.docx]

**Dylan Pashley** is a research assistant at the Department of Sociology at Lund University in Lund, Sweden. His interests include health care policy, transparent governance and data-driven methods.

**Piotr Ozieranski**is a senior lecturer at the Department of Social and Policy Sciences at the University of Bath, in the UK. He studies pharmaceutical policy, especially health technology assessment and drug reimbursement, as well as matters related to transparency and conflicts of interest.

**Shai Mulinari** is an associate professor and senior lecturer at the Department of Sociology at Lund University in Lund, Sweden. He undertakes research on pharmaceuticals use and regulation, health and health-care inequalities, and pharmaceutical industry practices, regulation, and transparency.
