# Supplementary material for: Disclosure of Pharmaceutical Industry Funding of Patient Organisations in Nordic Countries: Can Industry Self-Regulation Deliver on its Transparency Promise?
Source: Int J Health Serv. 2022 Mar 1;52(3):347–62. doi: 10.1177/00207314221083871 (PMC9203660; doi:10.1177/00207314221083871)
Supplement: sj-docx-5-joh-10.1177_00207314221083871 - Supplemental material for Disclosure of Pharmaceutical Industry Funding of Patient Organisations in Nordic Countries: Can Industry Self-Regulation Deliver on its Transparency Promise? [file sj-docx-5-joh-10.1177_00207314221083871.docx]

Supplementary Table 1

Availability, accessibility, and format of transparency reports in Norway (2017–2019)

| **Company^a^** | **Out-link name (translated)** | **Out-link to webpage with patient organization disclosure** | **Patient organization disclosure found** | **Format** | **Years available** |
| --- | --- | --- | --- | --- | --- |
| AbbVie | Transfers of value to HCP/HCO | Yes | Yes | PDF, national | 2017, 2018, 2019 |
| Amgen | Transfers of value to HCP/HCO | No | Yes | Searchable database, international | 2017, 2018, 2019 |
| Astellas | Transfers of value to HCP/HCO | No | Yes | Searchable database, international | 2017, 2018, 2019 |
| AstraZeneca | Transfers of value to  patient and user organizations | Yes | Yes | PDF, national | 2017, 2018, 2019 |
| BASF | Transfers of value to HCP/HCO | No | Only HCP/HCO | N/A | N/A |
| Bayer | Disclosure of transfers of value | No | Only HCP/HCO | N/A | N/A |
| Biogen | Transfers of value to HCP/HCO | Yes | Yes | Online, national | 2019 |
| Boehringer Ingelheim | Disclosure of transfers of value | Yes | Yes | PDF, national | 2018, 2019 |
| Bristol-Myers Squibb | Transfers of value to HCP/HCO | No | Yes | PDF, national | 2017, 2019 |
| Daiichi Sankyo Oncology | Disclosure of transfers of value | No | Only HCP/HCO | N/A | N/A |
| Eisai | Transfers of value to HCP/HCO | Yes | Yes | PDF, separately reported within HCO/HCP disclosure, national | 2018 |
| Eli Lilly | Transfers of value to HCP/HCO and to  patient and user organizations | Yes | Yes | PDF, national | 2017, 2018, 2019 |
| Fresenius Kabi | Transfers of value to HCP/HCO | No | No | N/A | N/A |
| GE Healthcare | Transfers of value to HCP/HCO | Yes | Yes, nothing to disclose | PDF, international | 2017, 2018, 2019^b^ |
| GlaxoSmithKline | Transfers of value to HCP/HCO | No | Yes | PDF, national | 2017, 2018, 2019 |
| Grünenthal | Disclosure of transfers of value | No | No | N/A | N/A |
| Janssen-Cilag | Disclosure of transfers of value | Yes | Yes | Searchable database, international | 2017, 2018, 2019 |
| LEO Pharma | Disclosure of transfers of value | No | Only HCP/HCO | N/A | N/A |
| Lundbeck | Disclosure of transfers of value | No | Only HCP/HCO | N/A | N/A |
| Merck | Disclosure of transfers of value | Yes | Yes | PDF, international | 2017, 2018, 2019 |
| MSD | Disclosure of transfers of value | Yes | Yes | PDF, national | 2017, 2018, 2019 |
| Mundipharma | Disclosure of transfers of value | No | Only HCP/HCO | N/A | N/A |
| Mylan | Disclosure of transfers of value | No | Only HCP/HCO | N/A | N/A |
| Navamedic | Disclosure of transfers of value | No | Only HCP/HCO | N/A | N/A |
| Novartis | Disclosure of transfers of value | No | Yes | PDF, international | 2017, 2018, 2019 |
| Novo Nordisk | Disclosure of transfers of value | Yes | Yes | Other, national | 2019 |
| Orion | Disclosure of transfers of value | No | Yes, nothing to disclose | PDF, international | 2017, 2018, 2019^2^ |
| Otsuka | Disclosure of transfers of value | No | Yes | PDF, national and international | 2017 |
| Pierre Fabre | Disclosure of transfers of value | No | Only HCP/HCO | N/A | N/A |
| Pfizer | Transfers of value to HCP/HCO and to  patient and user organizations | Yes | Yes | PDF, national | 2017, 2018, 2019 |
| Roche | Disclosure of transfers of value | Yes | Yes | Searchable database, international | 2019 |
| Sandoz | Disclosure of transfers of value | Subsidiary of Novartis; link to Novartis page | No | PDF, international (Novartis) | N/A |
| Sanofi | Transfers of value to HCP/HCO | No | Yes | Online, national | 2019 |
| Shire | Disclosure of transfers of value | Yes; acquired by Takeda; link to Takeda page | Yes | PDF, under separate heading of Takeda disclosure, national | 2019 |
| Takeda | Disclosure of transfers of value | Yes | Yes | Online, national | 2017, 2018, 2019 |
| UCB | Disclosure of transfers of value | No | Yes | Searchable database, PDF, international | 2017, 2018, 2019 |

^a^ Company list based on Norwegian PTA gateway.

^b^ Company reportedly made no payments in these years.

Supplementary Table 2

Availability, accessibility, and format of transparency reports in Finland (2017–2019)

| **Company^a^** | **Out-link name** | **Out-link to webpage with patient organization disclosure** | **Patient organization disclosure found** | **Format** | **Years available** |
| --- | --- | --- | --- | --- | --- |
| AbbVie | Company name | No | Yes | PDF, national | 2017, 2018 |
| Amgen | Company name | No | Yes | PDF, national | 2017, 2018, 2019 |
| Astellas | Company name | No | Yes | Searchable database, international | 2019 |
| AstraZeneca | Company name | No | Yes | PDF with HCP/HCO, national | 2017, 2018, 2019 |
| Bayer | Company name | No | Yes | PDF, national | 2017, 2018, 2019 |
| Berlin-Chemie | Company name | N/A | Not found | N/A | N/A |
| Biocodex | Company name | N/A | Not found | N/A | N/A |
| Biogen | Company name | Yes | Yes | Online, national | 2017, 2018, 2019 |
| Boehringer Ingelheim | Company name | No | Yes | PDF with HCP/HCO, national | 2017, 2018, 2019 |
| Bristol-Myers Squibb | Company name | N/A | Not found | N/A | N/A |
| Celgene | Company name | N/A | Not found | N/A | N/A |
| Eli Lilly | Company name | No | Yes | PDF, national | 2017, 2018, 2019 |
| Ferring | Company name | Yes | Yes | Online, national | 2019 |
| GlaxoSmithKline | Company name | No | Yes | Online, national | 2019 |
| Janssen-Cilag | Company name | Yes | Yes | Searchable database, international | 2017, 2018, 2019 |
| LEO Pharma | Company name | Yes | Yes | Online, national | 2018, 2019 |
| Lundbeck | Company name | Yes | Yes | PDF, national | 2017, 2018, 2019 |
| Merck | Company name | Yes | Yes | PDF, international | 2017, 2018, 2019 |
| MSD | Company name | No | Yes | PDF, international | 2018, 2019 |
| Novartis | Company name | No | Yes | PDF, international | 2017, 2018, 2019 |
| Novo Nordisk | Company name | N/A | Not found | N/A | N/A |
| Pfizer | Company name | Yes | Yes | Online, national | 2017, 2018, 2019 |
| Roche | Company name | Yes | Yes | Searchable database, international | 2019 |
| Sanofi | Company name | No | Not found | N/A | N/A |
| Santen | Company name | No | Not found | N/A | N/A |
| Servier | Company name | N/A | Not found | N/A | N/A |
| Shire | Company name | Yes, acquired by Takeda; link to Takeda page | Yes | PDF, under separate heading of Takeda disclosure, national | 2019 |
| Sobi | Company name | No | Yes | Online, national | 2017, 2018, 2019 |
| Takeda | Company name | Yes | Yes | Online, national | 2019 |
| UCB | Company name | No | Yes | Searchable database, PDF, international | 2017, 2018, 2019 |

^a^ Company list based on Finnish PTA gateway.
